# Could low grade bacterial infection contribute to low back pain? A systematic review

**DOI:** 10.1186/s12916-015-0267-x

**Published:** 2015-01-22

**Authors:** Donna M Urquhart, Yiliang Zheng, Allen C Cheng, Jeffrey V Rosenfeld, Patrick Chan, Susan Liew, Sultana Monira Hussain, Flavia M Cicuttini

**Affiliations:** Department Epidemiology and Preventive Medicine, School of Public Health and Preventive Medicine, Monash University, Alfred Hospital, Commercial Road, Melbourne, Victoria 3004 Australia; Department of Surgery, Central Clinical School, Monash University, Alfred Centre, Commercial Road, Melbourne, Victoria 3004 Australia; Department of Neurosurgery, Alfred Hospital, Commercial Road, Prahran, Victoria 3181 Australia; Department of Orthopaedic Surgery, Alfred Hospital, Commercial Road, Prahran, Victoria 3181 Australia

**Keywords:** Bacteria, Disc, Infection, Low back pain, Modic change, Systematic review

## Abstract

**Background:**

Recently, there has been both immense interest and controversy regarding a randomised, controlled trial which showed antibiotics to be effective in the treatment of chronic low back pain (disc herniation with Modic Type 1 change). While this research has the potential to result in a paradigm shift in the treatment of low back pain, several questions remain unanswered. This systematic review aims to address these questions by examining the role of bacteria in low back pain and the relationship between bacteria and Modic change.

**Methods:**

We conducted electronic searches of MEDLINE and EMBASE and included studies that examined the relationship between bacteria and back pain or Modic change. Studies were rated based on their methodological quality, a best-evidence synthesis was used to summarise the results, and Bradford Hill’s criteria were used to assess the evidence for causation.

**Results:**

Eleven studies were identified. The median (range) age and percentage of female participants was 44.7 (41–46.4) years and 41.5% (27–59%), respectively, and in 7 of the 11 studies participants were diagnosed with disc herniation. Nine studies examined the presence of bacteria in spinal disc material and all identified bacteria, with the pooled estimate of the proportion with positive samples being 34%. *Propionibacterium acnes* was the most prevalent bacteria, being present in 7 of the 9 studies, with median (minimum, maximum) 45.0% (0–86.0) of samples positive. The best evidence synthesis found moderate evidence for a relationship between the presence of bacteria and both low back pain with disc herniation and Modic Type 1 change with disc herniation. There was modest evidence for a cause-effect relationship.

**Conclusions:**

We found that bacteria were common in the spinal disc material of people undergoing spinal surgery. There was moderate evidence for a relationship between the presence of bacteria and both low back pain with disc herniation and Modic Type 1 change associated with disc herniation and modest evidence for causation. However, further work is needed to determine whether these organisms are a result of contamination or represent low grade infection of the spine which contributes to chronic low back pain.

## Background

There has been both immense interest and controversy regarding a recent randomised, controlled trial (RCT) which showed antibiotic treatment to be effective in the treatment of chronic low back pain in individuals with herniated discs and associated Modic Type 1 changes (bone oedema) on magnetic resonance imaging (MRI) [1]. The RCT was based on the hypothesis that some individuals with a disc herniation develop chronic low back pain due to a secondary infection that occurs in the disc. While this research has the potential to result in a paradigm shift in the treatment of low back pain, it has not currently been translated into clinical practice. These findings have some similarities to the discovery of *Helicobacter pylori* and the shift it led to in the way peptic ulcers are treated. However, a greater understanding of the evidence underlying this RCT is required before a change in practice can be justified. Moreover, although the potential for good is high, the potential for harm in selecting for antimicrobial resistance with the prolonged use of broad spectrum antibiotics is also significant.

This recent RCT by Albert et al. [1] was based on the findings of their previous biopsy study which suggested that those who developed early Modic Type 1 changes were more likely to have infection with *Propionibacterium acnes* (*P. acnes*) [2]. However, there are several questions that remain unanswered; have other studies investigated the presence of bacteria in people with low back pain, and if so, what bacteria were identified, what were the associated participant and clinical characteristics, what evidence is there that bacteria are associated with low back pain, and are Modic changes markers for bacterial infection. This systematic review aims to address these questions by examining the evidence for the presence of bacteria in spinal structures of patients undergoing lumbar spine surgery and investigating the relationship between bacterial infection and Modic change. This work has the potential to provide a greater understanding of the evidence behind a potentially effective and safe treatment approach for individuals with chronic low back pain and disc herniation, thus significantly reducing the individual suffering and societal burden associated with this condition.

## Methods

This systematic review was conducted according to the 2009 PRISMA statement [3,4].

### Search strategy

A computerised search strategy was performed using MEDLINE and EMBASE from January 1946 until March 2014. We used the following MeSH headings and key words; ‘bacteria’, ‘infection’, ‘propionibacterium acnes’, ‘microbial’, ‘low back pain’, ‘Modic change’, and ‘lumbar surgery’. The search was limited to human studies of adults published in the English language. We also searched the reference lists of studies included in this review.

We included studies which examined i) the role of bacteria in low back pain and ii) the relationship between the presence of bacteria and Modic changes. Studies where participants had low back pain secondary to a previous bacterial exposure or current systemic infection were excluded.

Information was extracted and tabulated on the characteristics of the identified studies and their cohorts, the prevalence and type of bacteria identified, the methods used for bacterial identification and to minimize contamination, the results of studies examining the relationship between bacteria and Modic change, and evidence for a causal relationship.

### Methodological quality

To assess the methodological quality of the included studies, two reviewers (DU and SMH) independently scored them using the adapted scoring system of Lievense et al. [5,6] (Table 1). Each of the items was scored as positive (1), negative (0), or unclear (?), with a maximum possible score of 100%. Where the reviewers disagreed and could not achieve consensus, a third reviewer (FC) gave a final judgement. High quality was defined as achieving a score above the mean of all quality scores.

**Table 1:** **Criteria used to assess the methodological quality of selected cohort and cross-sectional studies (Lievense et al.** [5,6]**)**

**Item**	**Criterion**	**Study type**
Study population		
1	Selection before disease was present or at uniform point	CH/CC/CS
2	Cases and controls were drawn from the same population	CC
3	Participation rate ≥80% for cases/cohort	CH/CC/CS
4	Participation rate ≥80% for controls	CC
5	Sufficient description of baseline characteristics	CH/CC/CS
Assessment of risk factor		
6	Presence of bacteria was blinded	CH/CC/CS
7	Presence of bacteria were measured identical for cases and controls	CC
8	Presence of bacteria were assessed prior to the outcome	CH/CC/CS
Assessment of outcome		
9	Low back pain/ Modic change was assessed identical in studied population	CH/CC/CS
10	Low back pain/ Modic change was assessed reproducibly	CH/CC/CS
11	Low back pain/ Modic change was assessed according to standard definitions	CH/CC/CS
Study design		
12	Prospective design was used	CH/CC/CS
13	Follow-up time ≥2 years	CH
14	Withdrawals ≤20%	CH
Analysis and data presentation		
15	Appropriate analysis techniques were used	CH/CC/CS
16	Adjusted for at least age and sex	CH/CC/CS

CH, Applicable to cohort studies; CC, Applicable to case–control studies; CS, Applicable to cross-sectional studies; OA, Osteoarthritis.

As the criteria specific to the methodological assessment of RCTs is not included in the study by Lievense et al. [5,6], the PEDro scale [7] was used. The PEDro scale rates 11 aspects of the methodological quality of RCTs as being absent or present (Table 2). The total score ranges from 0 to 10, as the first item (eligibility criteria) is not included in the scoring. Studies that obtain a score of less than 6 points are considered to be of low quality, while those with a score of greater than 6 points are of high quality.

**Table 2:** **The PEDro Scale** [7] **– criteria used to assess the methodological quality of selected randomised control trials**

	**Yes**	**No**	**Where/comments**
1. Eligibility criteria were specified			
2. Subjects were randomly allocated to groups (in a crossover study, subjects were randomly allocated an order in which treatments were received)			
3. Allocation was concealed			
4. The groups were similar at baseline regarding the most important prognostic indicators			
5. There was blinding of all subjects			
6. There was blinding of all therapists who administered the therapy			
7. There was blinding of all assessors who measured at least one key outcome			
8. Measures of at least one key outcome were obtained from more than 85% of the subjects initially allocated to groups			
9. All subjects for whom outcome measures were available received the treatment or control condition as allocated or, where this was not the case, data for at least one key outcome was analysed by “intention to treat”			
10. The results of between-group statistical comparisons are reported for at least one key outcome			
11. The study provides both point measures and measures of variability for at least one key outcome			
TOTAL (checked excluding eligibility criteria specified):			

### Best evidence synthesis

A best evidence synthesis was used to summarise the data (Table 3). It was not possible to perform a meta-analysis due to the heterogeneity between the studies. The studies were ranked according to their design, with cohort studies considered to be a higher level of evidence than case control and cross-sectional studies. The level of evidence of studies was determined in conjunction with the quality score calculated for each study.

**Table 3:** **Criteria list for determining the level of evidence for best evidence synthesis, adapted from Lievense et al.** [6,7]

**Level of evidence**	**Criteria for inclusion in best evidence synthesis**
Strong evidence	Generally consistent findings in multiple high quality cohort studies
Moderate evidence	Generally consistent findings in 1 high quality cohort study, >2 high quality case–control studies, or >3 high quality case–control studies
Limited evidence	Generally consistent findings in a single cohort study, 1 or 2 case–control studies, or multiple cross-sectional studies
Conflicting evidence	Inconsistent findings in <75% of the trials
No evidence	No studies could be found

### Bradford Hill’s criteria for causation

We used Bradford Hill’s criteria to examine the evidence for causation [8]. These criteria, along with a description of each, are included in Table 4. The Bradford Hill’s criteria are commonly used to determine whether there is adequate evidence of a causal relationship between an incidence and a consequence.

**Table 4:** **Evidence for a causal relationship between low virulent bacteria and low back pain according to Bradford Hill’s criteria**

**Bradford Hill’s criteria**	**Description of criterion**	**Evidence for a causal relationship between bacteria and low back pain**
**Temporal relationship**	This is an essential criterion. For a possible risk factor to be the cause of a disease it has to come before the disease. This is generally easier to establish from cohort studies but rather difficult to establish from cross-sectional or case–control studies when measurements of the possible cause and the effect are made at the same time.	There is one longitudinal study available [2]. However, this only examined the development of Modic changes in participants with disc herniation (with and without positive cultures).
**Plausibility**	The association of a risk factor with a disease is more likely to be the cause of the disease if the association found is consistent with knowledge obtained from other sources such as animal experiments, experiments on biological mechanisms, etc. However, this criterion must be used with care because, often, the lack of plausibility may simply reflect a lack of scientific knowledge.	It is plausible for low virulent bacteria to cause chronic infection and symptoms such as low back pain. However, the bacteria isolated are also known potential contaminants.
**Consistency**	If similar results have been found in different populations using different study designs, then the association is more likely to be causal since it is unlikely that all studies were subject to the same type of errors (chance, bias, or confounding). However, a lack of consistency does not exclude a causal association since different exposure levels and other conditions may reduce the impact of the causal factor in certain studies.	There is consistency in the results across a number of studies; however, these studies were largely all cross-sectional in design and in similar populations (i.e., those having spinal surgery for disc herniation). A study that examined a control group (consisting of patients with trauma, myeloma, scoliosis, and degenerative disc disease) found patients with low back pain (sciatica) to have more positive tissue cultures (53% [19/36]) as compared with controls (0% [0/14] *P* = 0.0003) (Stirling et al. [17]).
**Strength of an association**	The strength of an association is measured by the size of the relative risk. A strong association is more likely to be causal than is a weak association, which could more easily be the result of confounding or bias.	In most of the identified studies there were a significant proportion of bacteria but few compared this to control groups.
**Dose–response relationship**	Further evidence of a causal relationship is provided if increasing levels of exposure lead to increasing risks of disease.	There is limited evidence to support a dose–response relationship.
**Specificity**	If a particular exposure increases the risk of a certain disease but not the risk of other diseases, then this is strong evidence in favour of a cause-effect relationship. However, one-to-one relationships between exposure and disease are rare and lack of specificity should not be used to say that a relationship is causal.	Similar bacterial species have been isolated in all studies, but few examined control groups.
**Reversibility**	When the removal of a possible risk factor results in a reduced risk of disease, then the likelihood that this association is causal is increased. Ideally, this should be assessed by conducting a RCT. Unfortunately, for many exposures/diseases such RCTs are just not possible in practice.	A single randomised controlled trial by Albert et al. [1] demonstrated that antibiotic treatment was effective in the treatment of chronic low back pain of greater than 6 month’s duration occurring after a previous disc herniation (in conjunction with Modic type 1 changes).
**Coherence**	The suggested cause-effect relationship should essentially be consistent with the natural history and biology of the disease.	The relationship is consistent with the natural history and biology of an infective process.
**Analogy**	The causal relationship will be further supported if there are similarities with other (well-established) cause-effect relationships.	Low grade infection in other sites (albeit involving prosthetic joints) may present with subacute or chronic pain and swelling.

RCT, Randomised controlled trial.

## Results

**Figure 1:**
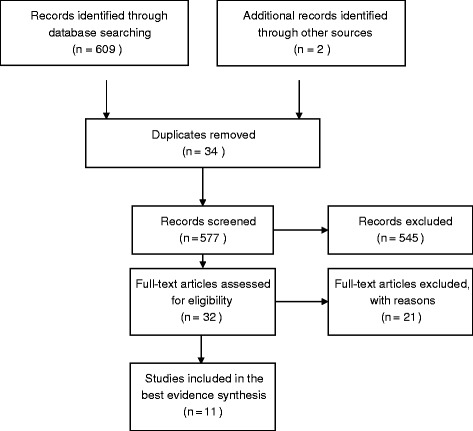
**PRISMA flow diagram showing the flow of information through the different phases of the systematic review.**

Eleven studies were included in this systematic review (Figure 1; Table 5). Of these, one was a RCT, one had a cross-sectional and a longitudinal component, and nine were cross-sectional. Ten studies examined the presence of bacteria in people undergoing spinal surgery (Table 6) and four studies investigated the relationship between the presence of bacteria and Modic changes (Table 7).

**Table 5:** **Characteristics of the 11 identified studies**

**Studies**	**Study design**	**Demographics, n (% Female)**	**Clinical features and surgery performed**	**Study inclusions/exclusions (includes previous interventions)**	**Modic changes examined**	**Control group**	**Quality score**
		**Mean age (years)**					
**Disc studies**							
Albert [2]	Cross-sectional/ cohort	61 (27% F)	Disc herniation	Inclusions:	Yes – Type, size, and volume were graded according to the Nordic Modic Protocol	No	78/75
		Age: 46.4	Primary surgery at a single spinal level	18 to 65 years old			
				MRI-confirmed lumbar disc herniation			
				Exclusions:			
				Received antibiotic treatment within previous 2 weeks			
Stirling [14]	Cross-sectional	36 (NA)	Discogenic radiculitis	Not specified	No	No	78
		Age: NA	Microdiscectomy				
Stirling [17]	Cross-sectional	207 (NA)	Discogenic radiculitis	Not specified	No	Yes – patients with trauma, tumour, or scoliosis	56
		Age: NA	Microdisectomy				
Agarwal [16]	Cross-sectional	52 (42% F)	Radiculopathy	Inclusions:	No	No	78
		Age: 43.9	Disc herniation	Sensory or motor symptoms in a single lumbar nerve distribution			
			Lumbar microdiscectomy				
				Positive physical examination findings (positive straight leg raise test, distributional weakness, diminished deep tendon reflexes)			
				MRI lumbar spine positive for HNP			
				Exclusions:			
				Diabetes mellitus, oral steroid use in the month before surgery, other immunosuppressive medications			
				Plain radiography demonstrating severe loss of disc height			
				High grade degenerative disc disease, spondylolisthesis > grade 1			
				History of prior lumbar surgery, multilevel symptomatic HNP or trauma			
				Red flags (progressive weakness, bowel/bladder complaints, radiographic unknown mass, unexpected weight loss)			
				Diagnosis of inflammatory arthritides, crystalline arthropathies, or other rheumatologic diseases			
Arndt [9]	Cross-sectional	83 (59% F)	Disc degeneration	Not specified	Yes – Type 1 and 2 according to the Modic classification	No	67
		Age: 41	Lumbar disc replacement at L3-4, L4-5, or L5-S1				
Coscia [11]	Cross-sectional	165 (NA)	Disc herniation	Not specified	No	Yes – Five groups, including cervical disc herniations, lumbar disc herniations, lumbar discogenic pain, deformity, and control	78
		Age: NA	Surgery not specified				
Ben-Galim [10]	Cross-sectional	30 (40% F)	Disc herniation	Exclusions:	No	No	67
		Age: 46.4 (NA)	Lumbar discectomy	Individuals who had been treated with antibiotics in the 2 months preceding the study			
				Those who had undergone back surgery			
				History of intradiscal injections			
Fritzell [12]	Cross-sectional	10 (40% F)	Disc herniation	Not stated	No	No	67
		Age (range): 20–47	Surgery not specified				
Carricajo [13]	Cross-sectional	54 (41%)	Disc herniation	Not stated	No	No	67
		Age: 44.8 (NA)	Surgery not specified				
Albert [1]	Randomised controlled trial	Intervention group:	Chronic LBP (>6 months) occurring after a previous disc herniation and who also had Modic type 1 changes in the vertebrae adjacent to the previous herniation	Inclusions:	Yes – Modic Type 1 only; size and volume of Modic changes were graded according to the Nordic Modic Protocol	Yes	100
				Aged between 18 and 65 years			
		90 (58.2% F)		MRI-confirmed disc herniation L3/L4 or L4/L5 or L5/S1 within the preceding 6–24 months			
		Age: 44.7 (10.3)					
		Placebo group:		Lower back pain of >6 months duration			
		72 (58.2% F)	Nil surgery	Modic type 1 changes adjacent to the previously herniated disc on repeat MRI			
		Age: 45.5 (9.2)		Exclusions:			
				Allergy to antibiotics			
				Current pregnancy or lactation			
				Any kidney disease			
				Pending litigation			
**Bone studies**							
Wedderkopp [15]	Cross-sectional	24 (58% F)	‘Persistent LBP’ Modic type I changes in at least 1 vertebra	Inclusion: Type 1 Modic changes on MRI	Yes – Modic Type 1 only	No	67
		Age: 43 (NA)					
			No surgery performed				

HNP, Herniated nucleus pulposus; LBP, Low back pain.

**Table 6:** **Methods used for bacteria identification and to minimize contamination and prevalence, and type of bacteria identified**

**Studies and design**	**Biopsy: Method, site and no. of specimens**	**Methods to minimize contamination**	**Duration of culturing biopsy material**	**Bacteria identification methods**	**Culture-positive samples (n, %)**	**Organisms identified in positive cultures (%)**	**Subsequently made generic analysis of** ***P. acnes*** **species**	**Quality score**
Albert [2] Cross-sectional	Open	All scalpels flamed before use as extra precaution	7 days with subsequent 1 day of subculture	Culture, PCR	28/61 (46%)	***P. acnes*** **: 86%**	Analytical profile index biochemical analysis using Rapid ID 32A kit (bioMerieux) and PCR amplification of 16S rDNA	78
	Disc material					Gram-positive cocci: 14%		
	Five specimens							
						Coagulase-negative **(**CN) staphylococci: 7%		
Stirling [14] Cross-sectional	Open	Stringent aseptic precautions taken to minimise risk of contamination	7 days	Culture, serology	19/36 (53%)	***P. acnes*** **: 84%**	Microscopy of Gram-stained smears of tissue samples	78
	Disc material					CN staphylococci: 11%		
	Not stated							
						Corynebacterium propinquum: 5%		
Stirling [17] Cross-sectional	Open	Not stated	7 days	Culture, serology	76/207 (37%)	***P. acnes*** **: 64%**	Microscopy of Gram-stained smears of tissue samples	56
	Disc material					CN staphylococci: 14%		
	Not stated					Propionibacteria: 10.5%		
Agarwal [16] Cross-sectional	Open	Disc material retained in a closed sterile sample cup	5 days	Culture	10/52 (19.2%)	***P. acnes*** **: 70%**	Not stated	78
	Disc material					Peptostreptococci: 10%		
	Not stated					Staphylococci aureus: 10%		
						CN staphylococci: 10%		
Arndt [9] Cross-sectional	Open	Disc structures stored in sterile syringes filled with physiological saline solution, care was taken to avoid contamination during conditioning process of biopsy	Blood agar, Drigalski agar: 24 h	Culture	40/83 (48.2%)	***P. acnes*** **: 45%**	Not stated	67
	Disc material		Polyvitex chocolate agar: 4 days			CN staphylococci: 40%		
	1 in 1st 25 disk replacements; 3 in following 58		Blood agar supplemented with hemin: 5 days			CN bacilli: 7.5%		
			Peptone glucose yeast broth: 10 days					
			Bactec Peds Plue bottle with fructooligosaccharide nutritional supplement: 7 days					
Coscia [11] Cross-sectional	Open	Specimens were obtained sterilely immediately at the time of surgical excision	Cultured using extended duration incubation techniques (repeated subcultures up to several weeks duration)	Culture	16/30 (53.3%)	Staphylococcus: 36%	Not stated	78
	Disc material					***P. acnes*** **: 18%**		
	Not stated							
Ben-Galim [10] Cross-sectional	Open	Samples are processed and cultured intraoperatively under stringent, sterile operating theatre conditions, culture mediums were warmed to room temperature before each operation	2 weeks	Culture	2/30 (6.7%)	CN staphylococci: 100%	Not stated	67
	Disc material							
	Four pieces (disc material dissected into four pieces)							
Fritzell [12] Cross-sectional	Open	Samples taken openly (no needle), all operations except for one were performed through a microscope with use of bipolar diathermy, assuring a very ‘dry’ operation field	Not applicable	PCR	(PCR)	(PCR)	Not stated	67
	Disc material				2/10 (20%)	Bacillus cereus: 50%		
	Two – one from annulus fibrosus and one from nucleus pulposus					Citrobacterbraaki/freundi: 50%		
Carricajo [13] Cross-sectional	Open	Obtained under aseptic conditions	One horse-blood agar, two chocolate PolyVitex agar: 10 days	Culture	12/54 (22%)	***P. acnes*** **: 17%**	Not stated	67
			One Schaedler medium: 20 days					
	Disc material, muscle, ligamentum flavum					Anaerobic streptococci: 8%		
	Three – muscle, ligamentum flavum, herniated intervertebral discs							
Wedderkopp [15] Cross-sectional	Needle	Obtained with sterile technique	2 weeks	Culture	2/24 (8.3%)	Staph epidermidis: 50%	Not stated	67
						CN staphylococci: 50%		
	Vertebral body							
	One – at site of Modic Type 1 change

**Table 7:** **Studies examining the relationship between the presence of low virulent bacteria and modic changes**

**Studies**	**Study design**	**Demographics N (% Female) Mean age (years)**	**Clinical features**	**Measure of modic change**	**Results**	**Quality score**
Albert [1]	Randomised controlled trial	Treatment group:	Chronic LBP (>6 months) occurring after a previous disc herniation and who also had Modic type 1 changes in the vertebrae adjacent to the previous herniation	Type, size, and volume graded according to the Nordic Modic Protocol	Modic changes: Treatment group: 142 (92.2%)	100
		90 (58.2% F)			Placebo group: 130 (97%)	
					Grade 1 Modic changes: Treatment group: 10.4%; Placebo group: 28.8%	
		Age: 44.7 (10.3)			*P* = 0.006	
		Placebo group:			At 1-year follow-up, 10 patients in both groups demonstrated no Modic changes	
		72 (58.2% F)				
					Treatment group: Significant decrease in volume; volume 2–4 were reduced to volume 1 (*P* = 0.05)	
		Age: 45.5 (9.2)			Placebo group: Not observed	
Albert [2]	Cohort	61 (27% F)	Disc herniation	Type, size, and volume graded according to the Nordic Modic Protocol	Discs (anaerobic bacteria): 80% developed new Modic changes in the vertebrae adjacent to the previous disc herniation. Discs (Aerobic): No new; MC discs (negative cultures): 44% new MC	78
		Age: 46.4				
					The association between an anaerobic culture and new MCs was significant	
					5.60 (95% CI 1.51–21.95), (*P* = 0.004)	
Arndt [9]	Cross-sectional	83 (59% F)	Disc degeneration	Modic changes (Type 1 and 2)	There was no significant association between Modic changes and positive cultures (*P* = 0.2)	67
		Age: 41				
Wedderkopp [15]	Cross-sectional	24 (58% F)	No clinical symptoms; Modic type I changes in at least 1 vertebra	Type 1 Modic changes only	There was no evidence of bacteria in vertebrae with Modic type 1 changes, with only 2/24 patients yielding bacteria.	67
		Age: 43 (NA)				

### What were the characteristics of people with low back pain that have bacterial infection?

Table 5 presents the characteristics of the study cohorts in the 11 identified studies. The average number of participants in the 11 studies was 74, with samples ranging from 10 to 207. The median (minimum, maximum) age and percentage of female patients was 44.7 (41, 46.4) years and 41.5% (27, 59), respectively. In five of the 11 studies, the participants were diagnosed with disc herniation alone [2,9-13], with the remaining six studies recruiting participants with discogenic radiculitis [14,15], radiculopathy and disc herniation [14,16,17], disc degeneration [10], disc herniation and Modic changes [1], and Modic changes only [15].

Three studies reported the use of a control group; Albert’s RCT included an intervention group receiving antibiotic treatment and a placebo group [1], Stirling et al. [17] recruited patients with trauma, tumours, or scoliosis for their controls, and, similarly, Coscia et al. [11] included patients with trauma or neuromuscular deformity. Four of the 11 studies specified the inclusion and exclusion criteria they used [2,10,16]; with previous use of antibiotics [2,10], allergy to antibiotics [1], and previous surgery [10,16] reported to be exclusions. None of the studies specified osteomyelitis or discitis as exclusion criteria.

### What was the prevalence and type of bacteria identified in studies examining participants undergoing spinal surgery?

**Figure 2:**
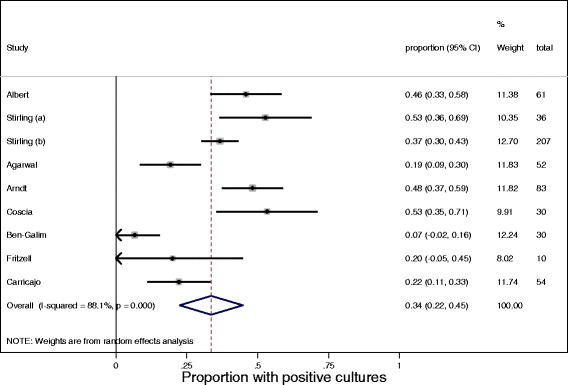
**Forest plot showing the proportion of positive cultures in the nine studies examining the presence of bacteria in disc material in patients undergoing spinal surgery.**

Table 6 presents the prevalence and type of bacteria identified in 10 studies examining participants undergoing spinal surgery. Nine studies investigated the presence of bacteria in spinal disc material, with one study examining the vertebral body with Modic Type 1 change. Of the nine studies that examined disc material, the median (minimum, maximum) percentage of culture positive samples was found to be 22.0% (6.7, 53.3). The data are presented as a forest plot (Figure 2) with the pooled estimate of the proportion with positive cultures being 34%. Eight of these nine studies identified more than one bacteria, with the study by Ben-Galim et al. [10] only reporting the presence of coagulase-negative staphylococci. While a variety of bacteria were identified, *P. acnes* was the most prevalent, being present in seven of the nine studies and the most common bacteria in six of the nine studies. A median (minimum, maximum) of 45% (0, 86.0) of samples were positive for *P. acnes*, followed by coagulase-negative staphylococci, which was present in 14% (0, 100) of cases. The study by Wedderkopp et al. [15], which investigated the presence of bacteria in the vertebral body, reported 8.3% of cultures to be positive, with staphylococcus epidermis and coagulase-negative staphylococci each being present in 50% of cultures.

There were a variety of methods used to identify bacteria and minimize contamination. Nine of the 10 studies used an open procedure to obtain biopsies, with one study by Wedderkopp et al. [15] using a closed, needle approach. Six studies used cultures to identify bacteria [9-11,13,15,16], two studies used culture and serology [14,17], and the remaining two used PCR with [2] or without culture [12]. Studies obtained between one and five specimens for assessment. The duration of culturing varied considerably between the studies, ranging from 5 to 7 days [4,10,14,15,17] to 2 to 3 weeks [11,12,16,18]. Moreover, only three of the 10 studies performed genetic analysis to determine the species of *P. acnes* identified [4,14,15]. With respect to minimizing contamination, five of the studies simply stated that aseptic or sterile techniques were used [10,11,13,15,17], while the others reported a wide variety of techniques including flaming scalpels [2], using bipolar diathermy [12], and collecting specimens in sterile cups and syringes [9,16].

### Are Modic changes markers of bacterial infection in low back pain?

Only four of the 11 studies we identified examined Modic changes. The RCT by Albert et al. [1] examined differences in Modic Type 1 changes after antibiotic treatment and found a reduction in their volume in the treatment group compared to the placebo group (*P* = 0.05). In Albert et al.’s biopsy study [2], low virulent bacteria were identified in participants who did and did not develop new Modic Type 1 changes, and conversely, those that had no bacteria present also developed Modic Type 1 changes. However, overall, a significant association between anaerobic bacteria and Modic Type 1 change was reported (5.60; 95% CI, 1.51–21.95; *P* = 0.004). The study by Arndt et al. [9] reported no significant association between the presence of bacteria and Modic Type 1 and 2 changes on MRI, and Wedderkopp et al. [15] reported that there was no evidence of bacteria in vertebrae with Modic Type 1 changes.

### What did the best evidence synthesis show with regards to the role of bacteria in low back pain and the relationship between bacteria and Modic changes?

Eleven studies examined the role of bacteria in low back pain (Table 5). This included one RCT and 10 cross-sectional studies. The mean methodological quality score of the 10 cross-sectional studies was 70%, with scores ranging from 56% to 78%. Four of the 10 observational studies were considered to be of high quality (according to the Lievense criteria), as they were given a quality score above the mean [2,11,14,16]. The RCT by Albert et al. [1] was also considered high quality as it scored greater than six on the PEDro scale. Overall, the best evidence synthesis indicated moderate evidence for a role of bacteria in low back pain with disc herniation.

Four studies examined the relationship between the presence of bacteria and Modic changes (Table 7). This included one RCT, one cohort study, and two cross-sectional studies. The RCT and cohort study were of high quality and the cross-sectional studies were of low quality. Overall, the best-evidence synthesis indicated moderate evidence for a relationship between the presence of bacteria and Modic Type 1 changes with disc herniation.

### What evidence is there for causation according to Bradford Hill’s criteria?

Table 4 presents Bradford Hill’s criteria, an explanation of each criterion, and evidence for a causal relationship between low virulent bacteria and chronic back pain in relation to each criterion. While there was evidence for causation with respect to five of Bradford Hill’s criteria (plausibility, strength of the association, specificity, reversibility, and coherence), there was no or limited evidence for the remaining four criteria (temporal relationship, consistency, dose–response relationship, and analogy). Overall, this approach suggests that there is modest evidence for a cause-effect relationship, with a major criteria (reversibility) being present between low virulent bacteria and chronic low back pain. However, further work is needed to clarify the role of bacterial infection in the development of low back pain with disc herniation.

## Discussion

This review identified 11 studies which examined the role of bacteria in individuals with low back pain. Ten studies reported the presence of bacteria in people undergoing spinal surgery, with *P. acnes* being the most commonly identified. Participants with bacterial infection were aged in their 40’s, were of both genders, and were most commonly diagnosed with lumbar disc herniation. The best evidence syntheses showed moderate evidence for low virulent bacteria having a role in low back pain with disc herniation and moderate evidence for a relationship between bacterial infection and Modic Type 1 change with disc herniation. There was also modest evidence for a causal relationship between the presence of bacteria and low back pain with disc herniation according to the Bradford Hill criteria.

All nine studies which examined the spinal disc material of individuals undergoing spinal surgery, found bacteria to be present in the disc, with pooled estimate of the proportion of positive cultures found to be 34%. *P. acnes* was the most common bacterium identified, being reported in seven of the nine studies, with a median (range) of 45.0% (0–86.0) of positive cultures. In contrast, the study by Wedderkopp et al. [15], which examined the presence of bacteria in the lumbar vertebrae, did not identify *P. acnes* in Modic Type 1 changes. While *P. acnes* is part of the normal human microbiota and has even been shown to stimulate protective responses against various cancers, there is growing evidence to suggest that it can have a pathological role in the human body, including being a cause of infections in injured structures and indwelling medical devices [18]. It is believed that the predominance of *P. acnes*, which is an anaerobic bacterium, may reflect the unusual environment in the disc where the lack of vascularity results in a very low oxygen tension and a low pH which provides ideal conditions for low virulent anaerobic bacteria to grow.

While each of the 10 studies identified bacteria in excised spinal tissue, there were inconsistencies between studies with respect to the prevalence of positive cultures and the types of bacteria identified. These inconsistencies may have been due to differences in study methodology, including the use of varying methods and time frames for detecting bacteria (cultures, PCR, and serology), culturing the biopsy material, and administering the antibiotic treatment. For instance, the two studies by Fritzell et al. [12] and Ben-Galim et al. [10], which did not report the presence of the *P. acnes*, used stringent methodology, including PCR to detect bacterial DNA and stringent aseptic biopsy methods, respectively, as well as longer time periods to culture the biopsies. Moreover, only six of the 10 studies reported on the administration of antibiotics, with three studies providing the dose before spinal tissue was excised [9,10,16] and three studies after tissue was removed [2,12,15].

Participants with positive cultures were of both genders, primarily in their 40’s, and most commonly presented for spinal surgery due to lumbar disc herniation. This clinical profile may be explained by the reduction in water content of the disc during the fifth decade and the greater susceptibility of the disc to injury, allowing influx of bacteria into the damaged disc and subsequent colonisation and chronic infection.

There was moderate evidence for a relationship between the presence of bacteria and Modic Type 1 changes with disc herniation. This evidence was based on the cohort study [2] and RCT [1] by Albert et al. that recruited low back pain participants with disc herniation and Type 1 Modic changes [2]. In contrast, the cross-sectional studies by Arndt [10] and Wedderkopp [15] found no association between bacteria and Type 1 and 2 Modic changes in people with disc degeneration and Type 1 Modic changes in those with ‘persistent’ low back pain, respectively. It is important to note that Modic Type 1 change and disc herniation are prevalent in 14 to 16% [19,20] and 30% (at the L5/S1 level) of individuals with low back pain, respectively [21]. Moreover, while the prevalence of disc pathology occurring concurrently with Modic Type 1 change has been reported to range from 11.5 to 17.5% [21], these data are not specific to disc herniation, and include other disc pathologies such as disc bulge and disc degeneration. However, overall, these results suggest that the concurrent prevalence of Modic Type 1 change and disc herniation is low and thus individuals with these pathologies, who may respond to treatments such as antibiotics, only represent a select subgroup of the low back pain population. Moreover, although we have focussed on the relationship between bacteria and Modic change, it has been hypothesised that Modic changes may also result from mechanical forces acting on the vertebral endplate. It is clear that further study is required to determine which clinical characteristics clearly identify the presence of bacteria in lumbar discs and the pathological processes involved with the development of Modic change.

The mechanism by which bacteria may enter the lumbar spinal tissue is unclear. There are several hypotheses presented in the literature. It has been suggested that injury to the disc which breaches the disc’s integrity allows low virulent organisms that are commonly present on human skin to travel via the blood stream to the disc [1,2]. The presence of the bacteria in the disc subsequently sets up an inflammatory response in the adjacent bone due to the release of cytokines and propionic acid, which enter the vertebrae through normal disc nutrition. It has also been suggested that the presence of bacteria may be the result of primary disc degeneration which allows pathogenic organisms to enter the disc and/or hinders their elimination [12]. Moreover, Arndt et al. [9] postulated that bacteria may be the result of haematogenous spread from a distant septic location, contiguous spread from an adjacent infection, or transvenous retrograde pathway from the pelvis. It is clear that further investigation is needed to understand the mechanisms underlying the presence of bacteria in spinal disc and bone material.

There is considerable debate in the literature about whether the presence of the bacteria in spinal discs is actually due to infection or a result of contamination during study procedures. While a number of studies in this review reported that the presence of bacteria might be due to low grade infection in the disc [2,11,12,14,16,17], several studies also suggested that contamination may play a role [10,13,15]. The inclusion of control cultures and control groups in future studies may assist in determining whether the low virulent bacteria identified are due to true infection or contamination.

There are several limitations and strengths to this study. There were a small number of studies identified and these were mainly cross-sectional and of modest quality. Only one RCT and one longitudinal study were identified. Studies varied considerably in their methodology, particularly in methods used to identify bacteria and minimize contamination and only three studies included a control group. A meta-analysis was not able to be performed due to the heterogeneity of the studies identified. However, the key strength of this study is that it is the first systematic review on this topic. We have performed a systematic search of the literature, tabulated the key features of the identified studies, performed a best evidence synthesis to summarise the data, and examined causation using the Bradford Hill criteria.

## Conclusions

This systematic review found moderate evidence to indicate low virulent bacteria have a role in low back pain with disc herniation and moderate evidence for a relationship between bacteria and Modic Type 1 change associated with disc herniation. While there was also modest evidence for causation, further work is needed to determine whether these low virulent organisms are a result of contamination or represent low grade infection of the lumbar spine which contributes to chronic low back pain associated with type 1 Modic changes in people with disc herniation.

## Abbreviations

LBP: Low back pain
*P. acnes*: *Propionibacterium acnes*
RCT: Randomized controlled trial
